# Imaging and Imaging-Based Management of Pediatric Thyroid Nodules

**DOI:** 10.3390/jcm9020384

**Published:** 2020-02-01

**Authors:** Ioannis Iakovou, Evanthia Giannoula, Christos Sachpekidis

**Affiliations:** 1 Academic Department of Nuclear Medicine, University Hospital AHEPA, School of Medicine, 54621 Thessaloniki, Greece; egiannoula@auth.gr; 2 Academic Department of Nuclear Medicine General Hospital PAPAGEORGIOU, 57006 Thessaloniki, Greece; 3 Clinical Cooperation Unit Nuclear Medicine, German Cancer Research Center (DKFZ), 69120 Heidelberg, Germany; christos_saxpe@yahoo.gr; 4 Department of Nuclear Medicine, Inselspital, Bern University Hospital, University of Bern, 3010 Bern, Switzerland

**Keywords:** thyroid nodules, thyroid cancer, children, imaging, ultrasound, elastography, fine needle aspiration biopsy (FNAB), scintigraphy

## Abstract

Thyroid nodules are less frequent in children than adults. Childhood thyroid nodules carry specific features, including a higher risk of malignancy than nodules in adults, rendering them unique in terms of management. Subsequently, they should be considered a distinct clinical entity with specific imaging recommendations. Initial evaluation requires a thorough workup, including clinical examination, and a detailed personal and familial history to determine the presence of possible risk factors. Laboratory and radiologic evaluation play an integral part in the diagnostic algorithm, with ultrasonography (US) being the first diagnostic test in all patients. US elastography has been recently introduced as an incremental method, reducing the subjectivity of the clinical diagnosis of nodule firmness associated with increased malignancy risk. However, fine-needle aspiration biopsy (FNAB) remains the mainstay in the diagnostic work-up of thyroid nodules and is documented to be best method for differentiating benign from malignant thyroid nodules. In addition, thyroid scintigraphy provides functional imaging information, which has a role both in the diagnostic management of thyroid nodules and during follow up in malignancies. Finally, despite providing additional information in certain clinical scenarios, **^18^**F-fludeoxyglucose Positron Emission Tomography (^18^F-FDG-PET), computed tomography (CT), and magnetic resonance imaging (MRI) imaging are not routinely recommended for the evaluation of patients with newly detected thyroid nodules or in all cases of thyroid cancer. The objective of this review is to summarize the concepts in imaging and imaging-based management of nodular thyroid disease in the pediatric population, acknowledging the unique features that this patient group carries and the specific approach it requires.

## 1. Introduction—Definitions

Thyroid nodules have been defined by the American Thyroid Association (ATA) as “discrete lesions within the thyroid gland, radiologically distinct from surrounding thyroid parenchyma” [1]. They may be discovered by palpation during a general physical examination or with imaging modalities performed for medical evaluations, such as ultrasound (US), computed tomography (CT) scans, magnetic resonance imaging (MRI) studies, or **^18^**F-fludeoxyglucose Positron Emission Tomography (**^18^**F-FDG-PET) scanning. The latter entities are called thyroid incidentalomas and they generally do not correspond to palpable thyroid lesions. Conversely, clinicians may identify palpable thyroid lesions that do not correspond to distinct radiological entities, and therefore would not be defined as thyroid nodules [2]. 

Compared to adults, thyroid tumors in children are larger and have a greater incidence of lymph node and lung metastases. Moreover, pediatric thyroid tumors are characterized by a higher recurrence rate. However, the overall prognosis for cancer death is much better in children.According to the latest ATA guidelines (GLs) on management for children with thyroid nodules and differentiated thyroid cancer (DTC), in order to more accurately define the impact of the physiological changes of growth and development on thyroid tumor behavior in this patient group, the upper age limit for pediatrics should be defined as patients ≤ 18 years of age, since the majority of children have completed development by this age [3]. 

## 2. Epidemiology

Both thyroid nodules and cancer are less common in children than adults. Nodule prevalence is estimated to be 0.2–5% in children compared with 19–35% in adults [4]. However, pediatric thyroid nodules have a higher likelihood of malignancy compared with those in adults (25% versus 10%, respectively) [5].

Relative to the common pediatric malignancies, such as leukemia, central nervous system (CNS) tumors, and lymphoma, thyroid cancer is rare in pediatric populations, affecting approximately 1 in every 1000 to 2000 children in the United States. However, it is the leading cause of pediatric endocrine cancer, accounting for over 6% of all pediatric cancers [6]. The incidence of thyroid cancer is increased in young adults, accounting for 13% of all invasive neoplasms [7].

Thyroid carcinomas in childhood are almost always well-differentiated. Recently, a multicentric study conducted on 120 pediatric patients with a thyroid nodule not associated with risk factors such as autoimmune thyroid diseases or radiotherapy revealed a 16% occurrence of thyroid carcinoma (73.7% papillary, 15.8% follicular, 10.5% medullary histotypes) [8].

According to the Surveillance Epidemiology and End Results (SEER) Cancer Registries, the estimated new cases of thyroid cancers in 2019 were 52,070 (1.8% of age <20 years old), while the estimated deaths attributed to the malignancy reached 2,170 (0.1% patients <20 years old) (https://seer.cancer.gov/statfacts/html/thyro.html, accessed on 27 January 2020). In Europe, an estimated 52,956 thyroid cancer (TC) cases were newly diagnosed in 2012, according to the latest records of the International Agency for Research on Cancer of the World Health Organization, (European Cancer). In the same year, 6336 Europeans were estimated to have died of TC (http://eco.iarc.fr/eucan/Cancer.aspx?Cancer=35, accessed on 12 December 2019). Thyroid cancer is more common in females than males. Based on the SEER registries, the age-standardized incidence rates (1987–1991) for thyroid cancer were 2.5 per 100,000 for males and 6.4 per 100,000 for females. Apart from this gender difference, racial variability is also observed, with Asians being more likely to be diagnosed with thyroid cancer, followed by whites and blacks [9]. In pediatric populations in particular, the National Cancer Institute’s SEER database was queried for all cases of pediatric thyroid cancer between the years 2007 and 2012 by Dermody et al. The authors showed that despite an annual increase in its incidence of approximately one percent, pediatric thyroid carcinoma remained an uncommon diagnosis. According to their results the average age-adjusted rate of malignancy was 0.59 per 100,000 patients, with a female to male ratio of 4:1 [10].

Contrary to Dermody’s results, Qian et al. showed that the increase of the annual percent change (APC) of pediatric thyroid carcinoma in the last years reached +4.6% between 1996 and 2016 [11]. The increase in incidence rates was similarly mirrored by increases in incidence rates for papillary histologic subtype, female sex, and adolescent age. Pediatric thyroid cancer incidence trends did not vary by tumor size or extent of disease. One important reason for this higher incidence of the malignancy is the steadily growing usage of more sensitive imaging techniques, as well the enhanced performance of fine-needle aspiration biopsies (FNAB), which enable the detection of small, indolent tumors, including incidental microcarcinomas sometimes of questionable clinical significance.

Besides their particular epidemiological characteristics, childhood thyroid nodules also carry some features which render them unique in terms of management: they seem to be affected differently by potential risk factors, they have other histological characteristics, they show frequent and precocious lymph node metastases when malignant as well as different clinical features compared with adults, and harbor specific molecular anomalies [11]. Such differences stress the particularity of the pediatric thyroid nodules, which should be subsequently considered a distinct clinical entity with specific imaging recommendations.

Having these in mind and considering the concerns raised by the continuously increasing usage of imaging techniques, which has led to unprecedented rates of incidental diagnosis of thyroid nodules; thus, raising the issue of potential overdiagnosis of pediatric thyroid cancer, we aim in this review article to present the main data published in the field of imaging of pediatric thyroid nodules.

## 3. Risk Factors for Thyroid Nodules and Factors Indicative of Higher Malignancy Risk

Risk factors for thyroid nodules in children include family history of thyroid nodules or thyroid cancer, iodine deficiency, radiation exposure, history of thyroid disease, elevated serum thyrotropin (TSH), gender, race, age, and several genetic syndromes (Table 1) [12,13,14].

Approximately 41% of children with thyroid nodules have a family history of thyroid disorders. Familial association is observed in 5% of all thyroid cancers with a follicular cell origin and 25% of those with parafollicular cell origin. Familial thyroid cancer from follicular cells is usually more aggressive than its sporadic form in terms of increased risk of recurrence and a younger age of onset. Familial non-medullary thyroid cancer may be associated with several syndromes, such as Carney’s complex (PPKAR1A gene mutation), Cowden’s syndrome (PTEN gene mutation), and familial adenomatous polyposis (FAP) (APC gene mutation). In particular, thyroid nodules are present in 67% of children presenting with Carney’s complex, and thyroid carcinoma is observed in 3.8% of patients. A total of 1–2% of patients with FAP, an inherited familial cancer characterized by an increased predisposition to colonic and extracolonic neoplasias, have thyroid carcinoma. Among childhood thyroid carcinomas of non-follicular origin, medullary thyroid cancer (MTC) is of familial origin in approximately 20% of patients. These cases are associated with a germline RET (ret proto-oncogene) mutation. Familial MTC may occur in isolation or as a part of multiple endocrine neoplasia (MEN) type II syndrome [15].

Thyroid nodules are often associated with other thyroid diseases, such as Hashimoto thyroiditis (HT), implying a causative relationship. A plethora of studies regarding the potential association between HT and thyroid cancer development have been carried out in adults. Most of them have demonstrated that the coexistence of HT and thyroid tumors—mainly papillary thyroid cancer (PTC)—is common, and that the risk of the development of thyroid cancer is significantly higher in patients with HT than in those without HT. Interestingly, HT seems to have a certain protective effect on the short- and long-term prognosis of cancer. In children, the available studies are few and with rather serious methodological limitations. However, similar to adults, the association between HT and thyroid cancer is also relatively common in children and adolescents. According to the results of these studies, the frequency of PTC in children and adolescents with HT is variable, from 0.7 to ~3%, while in patients with PTC the prevalence of coexisting HT varies from 6.3% to more than 40% of the cases. In a study including 108 pediatric thyroid cancer cases, HT was detected in 28.7% of the enrolled patients [16]. Furthermore, congenital hypothyroidism caused by dyshormonogenesis or by an iodine transporter defect increases the risk of nodules in children that can evolve to thyroid cancer of the follicular type [16].

A causal association between external radiation exposure and thyroid cancer is strongly supported by epidemiologic studies, since the young thyroid gland is particularly radiosensitive. Since 1995, the amount of information on thyroid cancer and childhood radiation exposure has increased substantially. Many of the original cohorts have extended their follow-ups and investigators have reported several new studies, resulting in a commensurate increase in the number of reported thyroid cancer cases. Previous studies showed an elevated risk for thyroid cancer in childhood cancer survivors who were treated for their primary malignancy with radiation therapy, especially Hodgkin lymphoma, leukemia, and central nervous system tumors [17,18]. However, smaller radiation doses were previously also used for non-cancer disorders in infants and children, such as enlarged thymus glands, enlarged tonsils, adenoids, and acne. Thyroid cancer from radiation exposure may occur as soon as 5–10 years after treatment and may persist for patients for 50 years or more after irradiation, with higher doses of radiation exposure seeming to be associated with an increased risk of cancer occurrence. Nevertheless, there is some variability in this result. Interestingly, high radiation doses were associated with a lower risk of malignancy, probably because high doses are likely to cause cell death. Besides radiation, chemotherapy drugs cause a four-fold increased risk of thyroid cancer [19,20].

Variations in population iodine intake are a primary determinant of benign thyroid disorders, such as goiters, nodules, and hyper- or hypothyroidism. Nowadays, it is clear that iodine deficiency sharply increases the risk of nodules and goiters in populations. However, the role of iodine intake in thyroid cancer remains uncertain, despite decades of study and debate. Several mechanisms linking iodine intake and thyroid cancer have been proposed, including chronic TSH stimulation, increase of H_2_O_2_-mediated, reactive oxygen species (ROS) generation, which can damage DNA and result in mutations, as well as associations with BRAF and RAS mutations [21]. 

Pediatric patients carrying such risk factors might benefit from prospective screening for thyroid nodules and thyroid cancer. According to the respective GLs, an annual physical examination is recommended in children at high risk for thyroid neoplasia, while additional imaging should be pursued if palpable nodules, thyroid asymmetry, and/or abnormal cervical lymphadenopathy are found on examination [3].

## 4. Initial Evaluation

A detailed personal and familial medical record to determine the presence of any of the aforementioned potential risk factors (positive family history, iodine deficiency, prior radiation exposure, history of thyroid disease, elevated TSH, gender, race, age, genetic syndromes) is mandatory, before physical, laboratory, and imaging examinations take place. Although physician-dependent, palpation gives the main idea about the location, size, consistency, and motility of the nodule. Despite the fact that there is no critical size for a nodule to be palpated, usually nodules >1.5 cm are palpable. Tenderness of the thyroid gland should be noted, as well as pain, changes over time, growth, and possible fixation to surrounding structures. In general terms, a large hard nodule, especially one adherent to adjacent tissue, is concerning for cancer [22]. As mentioned above, an annual physical examination is recommended in children at high risk for thyroid neoplasia, potentially with additional imaging. Regarding patients at increased risk of developing familial DTC, they should be referred to specialized centers so that appropriate evaluation, follow-up, genetic counseling, and/or treatment can be undertaken without subjecting patients and families to unwarranted and aggressive treatments [3].

Laboratory evaluation of the thyroid nodule includes serum TSH, T3, T4, AntiTPO antibodies, and calcitonin measurements. Serum TSH should be measured during the initial evaluation of a patient with a thyroid nodule. A higher serum TSH level, even within the upper part of the reference range, is associated with increased risk of malignancy in a thyroid nodule, as well as a more advanced stage of thyroid cancer [23]. T3, T4, and antiTPO antibodies evaluation is not routinely indicated unless hyperthyroidism is suspected, while calcitonin should be measured in high-risk patients for MTC (known mutation). Finally, in cases of known malignancy Tg and basal calcitonin may serve as biochemical tumor markers used postoperatively for surveillance. Calcitonin stimulating tests with Ca**^2+^**or pentagastrin are very useful, both for excluding an MTC when basal calcitoninis in the grey zone (15–50 ng/L) and for detecting residual disease or recurrence after surgery for MTC in patients with low basal levels [15].

## 5. Ultrasonography (US)

Imaging of thyroid nodules plays an integral part of the diagnostic algorithm. Being noninvasive, inexpensive, and highly sensitive, US is recommended as the first diagnostic test in all children with thyroid nodules. The US report should convey nodule size and location and a description of the nodule’s sonographic features, including composition (solid, cystic proportion, or spongiform), echogenicity (hyper-, iso-, hypoechoic), margins (smooth, irregular, lobulated, ill-defined, Halo, extrathyroid extension), presence and type of calcifications, shape, if taller than wide, and vascularity. The pattern of sonographic features associated with a nodule confers a risk of malignancy and, combined with nodule size, guides FNAB decision-making [24]. In general, the US features most concerning for malignancy are hypoechogenicity, irregular margins, and increased intranodular blood flow, as well as the presence of microcalcifications and abnormal cervical lymph nodes. On the other hand, entirely cystic nodules are regarded as almost always benign [3,25,26].

Notably, apart from the greater probability of DTC (22–26%) in pediatric populations, differences exist between children and adults regarding the frequency of DTC subtypes. For example, children and adolescents may have a widely invasive form of PTC called diffuse sclerosing variant PTC, which has abundant microcalcifications on US but usually not discrete thyroid nodules. For such reasons, in children, US-guided FNAB should be performed in all thyroid nodules of more than 10 mm, unless purely cystic, and in thyroid nodules 5–10 mm with suspicious US features. FNAB of suspicious lymph nodes with a thyroglobulin measured on the needle washout should also be performed prior to performing a lateral neck lymph node dissection [4].

Several studies have investigated the sonographic features of malignant pediatric thyroid nodules [8,25,26,27,28,29]. In the largest and most detailed series, including 404 nodules in 314 children, Richman et al. showed that most of the sonographic characteristics of a nodule associated with increased risk of cancer in adults also convey a higher risk of malignancy in children. In particular, the presence of speckled calcifications, irregular margins, taller-than-wide shape, and abnormal lymph nodes conveyed an increased cancer risk. Moreover, similarly to adults, solid nodules as well as solitary nodules were more likely to be malignant. On the contrary, unlike adults, in whom size is not associated with increased malignancy risk, in their series the malignancy rate increased with increasing nodule size. In addition, patients with an abnormal background sonographic appearance documented a higher risk of malignancy as compared to patients with normal background gland echo texture. Furthermore, certain US characteristics, including a taller-than-wide shape, presence of calcifications, and presence of abnormal lymph nodes, showed fairly high specificity but only moderate sensitivity for malignancy, confirming the—documented in adults—insufficiency of sonographic evaluation alone in enabling differentiation between benign and malignant thyroid nodules. Interestingly, with regards to color Doppler analysis, it was found to be not a useful differentiating characteristic in the identification of thyroid cancer [30].

ATA’s panel does not recommend US screening of the thyroid even in children carrying some factors associated with higher malignancy risk, such as those with a history of radiation exposure to the thyroid. The main reason for this is that the impact of the detection of subclinical disease by US prior to a palpable abnormality on the long-term outcome of these patients is not clear. On the other hand, evaluation by an experienced thyroid ultrasonographer should take place in children with autoimmune thyroiditis and a suspicious thyroid examination (suspected nodule or significant gland asymmetry), especially if associated with palpable cervical lymphadenopathy [3]. The sonographic patterns of thyroid nodules and theirestimated risk of malignancy as well as the main differences between pediatric and adult thyroid nodules are concisely described in Table 2.

## 6. US Elastography

Elastography is a novel, noninvasive imaging technique for the US evaluation of the mechanical properties (elasticity) of different types of tissue. Particularly in thyroid nodule diagnostics, US elastography was introduced as a method reducing the subjectivity of the clinical diagnosis of nodule firmness associated with increased malignancy risk. In general, it acquires information on the movement of the tissue in response to the application of a small amount of pressure: in softer tissues, the applied pressure causes the tissue to compress more, whereas harder tissues compress less [31].

Two methods are used for determining nodule stiffness: strain elastography, in which force is applied to the tissue by manual compression and the tissue deformation is parallel to the direction of the force, and shear-wave elastography (SWE), in which a push beam is created and the tissue deformation is perpendicular to the direction of the force [32]. Colors around and within the nodules are evaluated and visually scored according to a 4- to 5-scale scoring system, the regions of interest being specified as the target lesion and the adjacent reference region. The strain ratio is calculated automatically, and a higher strain ratio translates to a higher probability of malignancy. Another form of evaluating the elastography data is by calculating the thyroid strain ratio to perform an objective, semi-quantitative analysis of the tissue stiffness [33].

Despite the increasing number of studies assessing the diagnostic value of elastography in adults, only a few of them focus on children with thyroid nodules, mainly due to specific problems concerning the use of the technique in the pediatric population, since the compressive nature of elastography induces discomfort in children [26]. Furthermore, the rarity of pediatric thyroid nodules in general causes a shortage in the study population needed to truly determine the importance of US elastography [34]. Notably, in a study by Rago et al., elastography could increase the accuracy of US in thyroid nodule differentiation regardless of the nodule size [35]. 

Importantly, in recent years the efficacy of elastography has been investigated in the thyroid gland of healthy subjects as well as of children with several thyroid pathologies. Yurttutan et al. assessed a baseline strain index for healthy pediatric thyroid tissue and determined a mean value of 0.54 ± 0.38 m/s for normal pediatric thyroid tissue relative to adjacent soft tissue at the same depth, with no significant difference between subjects on the basis of sex, age, weight, height, or body mass index [36]. BakırtaşPalabıyık et al. measured the elasticity of thyroid tissue in children and adolescents using SWE and investigated the role of SWE in the diagnosis of autoimmune thyroiditis in childhood. Quantitative elastographic analysis evaluated by SWE in autoimmune thyroiditis patients (3.7 ± 1.2 m/s) was significantly higher compared with normal pediatric patients (1.8±0.3 m/s) and the optimal cut-off value was 2.39 m/s [37]. In parallel, with their recent study Cunha et al. evaluated the usefulness of strain elastography as an additional diagnostic tool for children who present with thyroid nodules. The authors found that the high elasticity of a nodule on elastography was associated with a low risk of thyroid cancer [33]. In agreement with them, Borysewicz-Sanczyk et al. found elastography to have a high negative predictive value for a benign diagnosis, by analyzing a total of 62 thyroid nodules in 47 children and adolescents [38]. Such studies suggest that the method may prove useful as a complementary test for screening thyroid nodules in children.

## 7. Fine-Needle Aspiration Biopsy (FNAB)

FNAB of a thyroid nodule is the best method for differentiating benign from malignant thyroid nodules and diffuse goiters. FNAB has long been the mainstay in the diagnostic work-up of adult thyroid nodules, and recent literature supports its efficacy in the pediatric population, with reported accuracy, sensitivity, and specificity of 99%, 94%, and 100%, respectively, per one recent study [39]. FNAB is a minor outpatient procedure performed in awake, non-fasting patients with appropriate aseptic technique, while local anesthesia is optional. FNAB can also be useful to evacuate cystic lesions, although the fluid will recollect in one-half of these cysts. To address terminology and other issues related to thyroid FNA, the National Cancer Institute (NCI) hosted the NCI Thyroid FNA State of the Science Conference. The conclusions regarding terminology and morphologic criteria from the NCI meeting led to the Bethesda Thyroid Atlas Project and formed the framework for The Bethesda System for Reporting Thyroid Cytopathology (TBSRTC). For clarity of communication, TBSRTC recommends that each report begin with one of six general diagnostic categories (Table 3) [40].

Although there is significant variability between studies, the risk of malignancy in non-diagnostic (Bethesda I), benign (Bethesda II), and suspicious/malignant (Bethesda V/VI) mirrors adult risk assessment at 0% (range 0–10%), 5–8% (range 0–16%), and 100%, respectively (Bethesda V range: 40–100%, Bethesda VI range: 100%). Several studies suggest that Bethesda III and IV categories account for up to 40% (range: 13–43%) of all pediatric FNAB diagnoses. Furthermore, these indeterminate aspirate diagnoses are associated with increased risk of malignancy in children versus adults (28% in children versus 6–30% in adults for atypia or follicular lesion of undetermined significance (AUS/FLUS) and 58% in children versus 10–40% in adults for follicular/Hürthle neoplasm or suspicious for follicular/Hürthle neoplasm (FN/SFN lesions). However, more recent studies indicate lower percentages of indeterminate FNAB groups than previously reported (13–18% for indeterminate diagnoses combined) and a lower risk of malignancy after histologic resection within these groups (11–20% for AUS/FLUS; 25–28% for FN/SFN) [6].

Cherella et al. investigated the differences in thyroid nodule cytology and malignancy risk between children and adults and found that pediatric nodules were more likely to be malignant than adult ones (19% versus 12%). Within cytological categories, malignancy rates were higher in pediatric nodules than in adult nodules that were cytologically non-diagnostic, atypia of undetermined significance (44% versus 22%), or suspicious for follicular neoplasm (71% versus 28%). No significant differences were observed between children and adults in the types of thyroid cancers diagnosed in these cytological categories. Malignancy rates did not differ between children and adults among nodules with cytology suspicious for papillary carcinoma or positive for malignancy. In this respect, their findings provide pediatric-specific data to inform the optimal management of thyroid nodules in children, which may differ from that of adult nodules with equivalent cytology [41].

As far as the performance of FNAB in children is concerned, ATA GLs recommend the same evaluation and treatment of thyroid nodules in children as in adults, with the following exceptions: (a) for the identification of nodules that warrant FNA, US characteristics and clinical context should be applied rather than size alone, (b) FNA should be performed under US guidance in children, (c) preoperative FNA of a hyperfunctioning nodule in a child is not warranted as long as the lesion is removed, (d) a diffusely infiltrative form of PTC may occur in children and should be considered in a clinically suspicious gland, and (e) for most nodules with indeterminate cytology, surgery is favored over repeat FNAB [3].

## 8. ^123^Ι/^131^Ι/^99m^Tc Thyroid Scintigraphy

Thyroid scintigraphy provides functional imaging of the gland based on the fact that thyroid follicular cells concentrate iodine efficiently. Both ^99m^Tc-pertechnetate (^99m^TcO4-) and iodine-123/131 (^123^I and ^131^I) can be used for thyroid imaging. Iodine is taken up by thyroid follicular cells via the sodium-iodine symporter (NIS), is organified and then incorporated into thyroid hormones. ^99m^TcO4-, on the other hand, is taken up by the thyroid but is not organified, and is thus not ideal for thyroid uptake calculations. Evaluation of pediatric thyroid nodules via radioiodine or ^99m^TcO4-thyroid scintigraphy has largely been replaced by high resolution US. Nevertheless, according to the European Association of Nuclear Medicine/Society of Nuclear Medicine and Molecular Imaging (EANM/SNMMI )practice guideline procedure standard for radioiodine uptake (RAIU) and thyroid scintigraphy, scintigraphy still has a role in evaluating the following: function of thyroid nodules detected on clinical examination and/or other imaging examinations classifying them into hyper-, iso-, or non-functioning nodules, multinodular goiter to identify suspicious hypofunctional “cold” areas that require further evaluation with FNAB or hyperfunctional “hot” thyroid nodules prior to radioiodine ablation [42]. Briefly, hyperfunctioning nodules do not require further cytologic evaluation, since they rarely harbor malignancy; nevertheless, indeterminate cytology (e.g., Bethesda classes III and IV, see below) is frequently reported in these cases. However, patients with overt or subclinical hypothyroidism as well as those with serum TSH, within the upper part of the reference range require further evaluation [23]. According to the ATA GLs, thyroid scintigraphy should be pursued for pediatric patients with a suppressed TSH associated with a thyroid nodule. Increased uptake within the nodule is consistent with autonomous nodular function and thyroid scintigraphy is the only examination able to demonstrate the presence of autonomously functioning thyroid nodules [3]. 

## 9. PET/CT

^18^FDG-PET imaging is not recommended for the evaluation of patients with newly detected thyroid nodules or thyroidal illness. Nonetheless, a focal or diffuse incidental detection of abnormal thyroid uptake may be encountered. In particular, focal ^18^F-FDG-PET uptake in the thyroid is incidentally detected in 1–2% of adult patients, while an additional 2% of patients demonstrate diffuse thyroid uptake [24]. Moreover, ^18^F-FDG-PET imaging is not routinely recommended for the evaluation of thyroid nodules with indeterminate cytology, despite the fact that several studies in the adult population have been published, examining the role of ^18^F-FDG-PET in the preoperative assessment of such nodules [43,44,45,46]. In a meta-analysis including seven studies, the sensitivity and specificity of ^18^F-FDG-PET were 89% and 55%, respectively, resulting in a 41% positive predictive value (PPV) and 93% negative predictive value (NPV), which is similar to the performance of the Gene Expression Classifier (GEC) [47]. Vriens et al. performed a cost-effectiveness analysis using ^18^F-FDG-PET performance data from their own meta-analysis and 2012 reimbursement rates of the Dutch system. They showed that ^18^F-FDG-PET was more cost-effective than surgery, the GEC, or mutational testing [48]. Recently, a prospective analysis of 87 adult patients who were scheduled to undergo surgery for thyroid nodule with indeterminate cytology used ^18^F-FDG-PET, multiparametric neck ultrasonography (MPUS), and ^99m^Tc-sestamibi (MIBI) scan to further evaluate thyroid nodules. The authors demonstrated that the accuracy of ^18^F-FDG-PET/CT in detecting thyroid malignancy is higher than that of ^99m^Tc-MIBI-scan and MPUS. According to their results, a negative ^18^F-FDG-PET/CT correctly predicts benign findings on histopathology. The association of FDG+/MPUS+ is significantly more specific than ^18^F-FDG-PET/CT alone in identifying DTC. A positive ^18^F-FDG-PET/CT is significantly associated with malignancy when the qualitative ^99m^Tc-MIBI-scan is rated as negative [49]. However, the modality cannot be routinely recommended in the care of children who have persistent evidence of DTC on follow-up [3]. Moreover, PET/CT with tracers other than ^18^F-FDG, such as ^124^I, despite its better physical characters,or the novel hybrid imaging modality PET/MRI, have not yet been compared with ^131^I scintigraphy and SPECT/CT in a large series of patients with DTC. These approaches are not yet widely available for clinical use and are considered to be primarily research tools at this time [24].

## 10. ^99m^Tc-sestamibi (MIBI) Scan 

It is not unusual that histopathological analysis cannot accurately discriminate benign from malignant lesions, since the detection or exclusion of capsular and/or vascular invasion cannot be done on cytological specimens in follicular-patterned lesions. As the large majority of such lesions are benign, the risk of inappropriate thyroid surgery is significant. In an attempt to address this issue, some authors have reported on the role of ^99m^Tc-sestamibi (MIBI) in differentiating malignant from benign thyroid lesions in patients with non-diagnostic/indeterminate cytology [50]. At visual evaluation, MIBI has a very high NPV in excluding thyroid malignancies, but at the same time a quite low specificity and PPV, since a positive MIBI scan can be found both in malignant and benign lesions [51]. The diagnostic accuracy of the MIBI thyroid scan has been proven to increase with the usage of quantitative analysis approaches. In particular, Saggiorato first evaluated the semi-quantitative Retention Index (R.I.) for the radiotracer, by calculating the ratio between uptake within the nodule and the normal thyroid tissue uptake [52]. More recently, other researchers introduced another quantitative index for the estimation of the MIBI washout from the nodule, the Wash-Out Index (WOind). The main difference between the two parameters lies in the fact that for WOind calculations the uptake within normal thyroid tissue, for which washout is faster than that within nodules, is not taken into account. Quantitative MIBI scintigraphy by evaluating WOind is a useful tool and is documented to be a more accurate method in the management of thyroid nodules with indeterminate cytology [53]. To our knowledge there are no available studies with MIBI on population ≤18 years of age. Thus, the modality should be implemented with caution in the evaluation of the pediatric thyroid nodule.

## 11. CT and MRI

Although US is the primary imaging test for a palpable thyroid nodule or a known thyroid malignancy, thyroid abnormalities are frequently first discovered on other cross-sectional modalities of CT and MRI. A thyroid lesion may be seen as an incidental finding, or CT and MRI may be used for the initial evaluation of an unknown neck mass. Moreover, CT and MRI can play a role in the preoperative assessment and staging of a known thyroid malignancy, since they have been described to be effective imaging methods for evaluating for nodal metastases and assessing the invasion of adjacent anatomical structures. Furthermore, they may aid in the assessment of recurrence in the post-treatment neck as well as during follow-up [54], while they are useful in the evaluation of potential trachea compression and respiratory compromise. However, an important issue associated with the use of CT in children is radiation exposure. The increased application nowadays of multidetector row CT scans as well as multiphase CT examinations in pediatric medicine raise the issue of high administered doses to the thyroid gland, potentially increasing the risk of thyroid cancer. Moreover, the application of iodine-based contrast agents in order to increase the sensitivity of the modality leads to suppression of radioactive iodine thyroid uptake, thus postponing a potential radioiodine treatment. These facts practically exclude CT scanning from screening for thyroid abnormalities and eliminate its role in thyroid cancer’s diagnostic and surveillance algorithm [55]. 

Regarding MRI, an important advantage is its excellent soft tissue contrast and reduced teeth bracelet-related artifacts in the head and neck, as compared to CT and ^18^F-FDG-PET/CT, rendering it the primary imaging modality for soft tissue tumors assessment. MRI of the neck is mainly used for planning the surgical approach in head and neck cancer and thyroid cancer patients. Additionally, gadolinium-based MRI-contrast media avoids iodine contamination before curative radioiodine therapy. Furthermore, radiation exposure is not an issue with MRI. However, similar to CT, MRI is not recommended in the routine clinical workup of all thyroid cancer patients of a younger age [56].

## 12. Conclusions

Although thyroid nodules are less common in children than adults, they pose a higher likelihood of malignancy. Thus, once diagnosed, they necessitate an immediate systematic, multistep evaluation to define a precise characterization. Thyroid nodules at high risk should be evaluated by US and FNA to confirm or exclude DTC, and allow, when appropriate, for complete preoperative surgical planning. Low-risk nodules can be followed with serial US and re-interrogated by FNA, or removed if they grow or develop suspicious US features. As far as nodules at indeterminate risk are concerned, all features, including family and personal history, US characteristics, FNA cytology, and molecular testing, should be considered to develop an appropriate personalized strategy. The role of nuclear medicine on the management of thyroid nodules and cancer in children is vital. The ATA GLs (2015) represent the current optimal care for children and adolescents with thyroid nodules. Taking into consideration that the ATA GL’s task force acknowledged the lack of randomized double-blind controlled clinical trials, stating that most published data are from retrospective cohorts, potentially subject to investigator bias, the need for the conduction of updated guidelines dedicated to patients ≤18 years of age is imperative. 

## Figures and Tables

**Table 1:** Risk factors for thyroid nodules and factors indicative of higher malignancy risk.

Exposure to Radioactivity	Medical Exposure
	External Beam Radiation Therapy
	Radioactive Contamination
Genes’ mutations andrelated types of thyroid tumors	RET:*MTC*
	HRAS, KRAS, NRAS: *FTA, FTC, FVPTC, PDTC, ATC*
	PI3KCA: *FTA, FTC, ATC, PTC*
	AKT1: *Metastatic cancer*
	CTNNB1: *PDTC, ATC*
	TP53: *PDTC, ATC*
	PPKAR1A: *PTC, HCTC*
	IDH1: *FTC, FVPTC, PTC, ATC*
	ALK: *ATC*
	APC: *NMTC*
	EGFR: *PTC*
	BRAF^V600E^: *PTC, FVPTC, TCPTC,ATC*
	BRAF^K601E^: *FVPTC*
	PTEN (mutation): *FTCA, FTC, ATC, PTC*
	PTEN (deletion): *FTC*
	NDUFA13: *HCTC*
Benign thyroid diseases	Autoimmune thyroiditis, most commonly Hashimoto
	Goiter
	Grave’s disease
	Iodine consumption
Dietary and Metabolic factors	Obesity
	Lack of physical exercise
	Smoking
	Female sex
Gender hormones and reproductive function	Menstruation before the age of 12 or after 14 years
	Gestation
	Exogenous hormones
	Trace elements associated with volcanic activity
Environmental factors	Air pollutants
	Xenobiotics
	Viruses

ALK, anaplastic lymphoma kinase; APC, adenomatous polyposis coli; ATC, anaplas ticthyroid cancer; EGFR, epidermal growth factor receptor; FTA, follicular thyroid adenoma; FTC, follicular thyroid cancer; FVPTC, follicular-variant PTC; HCTC, Hürthle cell thyroid cancer; IDH1, isocitrate dehydrogenase 1; NDUFA13, NADH dehydrogenase (ubiquinone) 1α subcomplex 13; NMTC, non-medullary thyroid carcinoma; PDTC, poorly differentiated thyroid cancer; PTC, papillary thyroid cancer; TCPTC, tall-cell PTC.

**Table 2:** Characters of thyroid nodules and differences between children and adults thyroid nodules.

**A. Sonographic patterns of thyroid nodules and estimated risk of malignancy** [24]**.**		
**Sonographic Pattern**	**US Features**	**Estimated Risk of Malignancy**
High suspicion	Solid hypoechoic nodule or solid hypoechoic component of a partially cystic nodule with one or more of the following features: irregular margins (infiltrative, microlobulated), microcalcifications, taller-than-wide shape, rim calcifications with small extrusive soft tissue component, evidence of extrathyroidal extension	>70–90%
Intermediate suspicion	Hypoechoic solid nodule with smooth margins without microcalcifications, extrathyroidal extension, or taller-than-wide shape	10–20%
Low suspicion	Isoechoic or hyperechoic solid nodule, or partially cystic nodule with eccentric solid areas, without microcalcification, irregular margin or extrathyroidal extension, or taller-than-wide shape.	5–10%
Very low suspicion	Spongiform or partially cystic nodules without any of the sonographic features described in low, intermediate, or high suspicion patterns	<3%
Benign	Purely cystic nodules (no solid component)	<1%
**B. Differences between pediatric and adult thyroid nodules**		
Difference	Pediatric	Adults
Epidemiology [4,5]	Less common. Nodule prevalence: 0.2–5%	More common Nodule prevalence: 19–35%
	Higher likelihood of malignancy (25%)	Lower likelihood of malignancy (10%)
Histology/Stage [3]	Higher incidence of regional lymph node involvement, extrathyroidal extension, and pulmonary metastasis	Lower incidence of regional lymph node involvement, extrathyroidal extension, and pulmonary metastasis
Prognosis [11]	More favorable progression-free survival in children Mortality rate ~0.1% in patients aged < 20	Less favorable progression-free survival in adults Maximum mortality rate up to 27.4% in patients aged 75–84
Molecular [3]	Higher prevalence of gene rearrangements and a lowerfrequency of point mutations in the proto-oncogenes implicatedin PTC	Lower prevalence of gene rearrangements and a higherfrequency of point mutations in the proto-oncogenes implicatedin PTC
	BRAF mutations are the less common abnormality in children PTC	BRAF mutations are the most common abnormality in adult PTC (36–83% of cases)
	RET/PTC rearrangements are more common in PTC from children	RET/PTC rearrangements are less common in adult PTC
Sonographic characteristics [8,25,26,27,28,29]	The malignancy rate is increased with increasing nodule size	The nodule’s size is not associated with increased malignancy risk
	Color Doppler analysisis not a useful differentiating characteristic in the identification of thyroid cancer	Color Doppler analysis has incremental value in the identification of malignancies
	Patients with an abnormal background sonographic appearance documented a higher risk of malignancy	A higher risk of malignancy is not documented for patients with an abnormal background sonographic appearance
	Diffuse sclerosing variant PTC, with abundant microcalcifications is more common in children	Diffuse sclerosing variant PTC with abundant microcalcifications is less common in adults

**Table 3:** The Bethesda System for Reporting Thyroid Cytopathology (TBSRTC) [40].

I	Non-diagnostic or unsatisfactory	Cyst fluid only Virtually acellular specimen Other (obscuring blood, clotting artifact, etc.)
II	Benign	Consistent with a benign follicular nodule (includes adenomatoid nodule, colloid nodule, etc.) Consistent with lymphocytic (Hashimoto) thyroiditis in the proper clinical context Consistent with granulomatous (subacute) thyroiditis Other
III	Atypia or follicular lesion of undetermined significance (AUS/FLUS).	
IV	Follicular/Hürthle neoplasm or suspicious for follicular/Hürthle neoplasm (FN or SFN)	Specify if Hürthle cell (oncocytic) type
V	Suspicious for malignancy (SUSP)	Suspicious for papillary carcinoma Suspicious for medullary carcinoma Suspicious for metastatic carcinoma Suspicious for lymphoma Other
VI	Malignant	Papillary thyroid carcinoma Poorly differentiated carcinoma Medullary thyroid carcinoma Undifferentiated (anaplastic) carcinoma Squamous cell carcinoma Carcinoma with mixed features (specify) Metastatic carcinoma Non-Hodgkin lymphoma Other

## Abbreviations

^18^F-FDG-PET	^18^F fludeoxyglucose Positron Emission Tomography
ATA	American Thyroid Association
AUS/FLUS	Atypia or follicular lesion of undetermined significance
CT	Calsitonin
CNS	Central Nervous System
CT	Computed Tomography
DTC	Differentiated Thyroid Cancer
EANM	European Association of Nuclear Medicine
FNAB	Fine Needle Aspiration Biopsy
FN/SFN	Follicular/Hürthle neoplasm or suspicious for follicular/Hürthle neoplasm
GEC	Gene Expression Classifier
GLs	Guidelines
HT	Hashimoto Thyroiditis
MTC	Medullary Thyroid Cancer
MRI	Magnetic Resonance Imaging
MTC	Medullary thyroid carcinoma
MPUS	Multiparametric neck ultrasonography
MEN	multiple endocrine neoplasia
NCI	National Cancer Institute
NPV	Negative predictive value
PTC	Papillary thyroid cancer
PPV	Positive predictive value
RAIU	Radioiodine uptake
SWE	shear-wave elastography
SEER	Surveillance Epidemiology and End Results
SUSP	Suspicious for malignancy
TBSRTC	The Bethesda System for Reporting Thyroid Cytopathology
TC	Thyroid Cancer
Tg	Thyroglobulin
TFNAC	thyroid fine needle aspiration cytology
US	Ultrasound
WOInd	Wash-Out Index

## References

[1] Cooper D.S., Doherty G.M., Haugen B.R., Kloos R.T., Lee S.L., Mandel S.J., Mazzaferri E.L., McIver B., Pacini F., Schlumberger M. (2009). Revised American Thyroid Association management guidelines for patients with thyroid nodules and differentiated thyroid cancer. Thyroid.

[2] Popoveniuc G., onklaas J. (2012). Thyroid Nodules. Med. Clin. North Am..

[3] Francis G.L., Waguespack S.G., Bauer A.J., Angelos P., Benvenga S., Cerutti J.M., Dinauer C.A., Hamilton J., Hay I.D., Luster M. (2015). Management Guidelines for Children with Thyroid Nodules and Differentiated Thyroid Cancer. Thyroid.

[4] Bauer A.J., Francis G.L. (2016). Evaluation and management of thyroid nodules in children. Curr. Opin. Pediatr..

[5] Mussa A., De Andrea M., Motta M., Mormile A., Palestini N., Corrias A. (2015). Predictors of Malignancy in Children with Thyroid Nodules. J. Pediatr..

[6] Paulson V.A., Rudzinski E.R., Hawkins D.S. (2019). Thyroid Cancer in the Pediatric Population. Genes.

[7] Massimino M., Evans D.B., Podda M., Spinelli C., Collini P., Pizzi N., Bleyer A. (2018). Thyroid cancer in adolescents and young adults. Pediatr. Blood Cancer.

[8] Corrias A., Mussa A., Baronio F., Arrigo T., Salerno M., Segni M., Vigone M.C., Gastaldi R., Zirilli G., Tuli G. (2010). Diagnostic features of thyroid nodules in pediatrics. Arch. Pediatr. Adolesc. Med..

[9] Holmes L., Hossain J., Opara F. (2012). Pediatric Thyroid Carcinoma Incidence and Temporal Trends in the USA (1973–2007): Race or Shifting Diagnostic Paradigm?. ISRN Oncol..

[10] Dermody S., Walls A., Harley E.H. (2016). Pediatric thyroid cancer: An update from the SEER database 2007–2012. Int. J. Pediatr. Otorhinolaryngol..

[11] Qian Z.J., Jin M.C., Meister K.D., Megwalu U.C. (2019). Pediatric Thyroid Cancer Incidence and Mortality Trends in the United States, 1973–2013. JAMA Otolaryngol. Head Neck Surg..

[12] Giannoula E., Iakovou I., Chatzipavlidou V. (2015). Risk factors and the progression of thyroid malignancies. Hell J. Nucl. Med..

[13] Giannoula E., Gkantaifi A., Iakovou I. (2016). Radiation treatment of head and neck carcinomas as a risk factor for thyroid carcinomas. Hell J. Nucl. Med..

[14] Xing M. (2013). Molecular pathogenesis and mechanisms of thyroid cancer. Nat. Rev. Cancer.

[15] Guille J.T., Opoku-Boateng A., Thibeault S.L., Chen H. (2015). Evaluation and management of the pediatric thyroid nodule. Oncologist.

[16] Penta L., Cofini M., Lanciotti L., Leonardi A., Principi N., Esposito S. (2018). Hashimoto’s Disease and Thyroid Cancer in Children: Are They Associated?. Front. Endocrinol..

[17] Sklar C., Whitton J., Mertens A., Stoval M., Green D., Marina N., Greffe B., Wolden S., Robison L. (2000). Abnormalities of the thyroid in survivors of Hodgkin’s disease:data from the Childhood Cancer Survivor Study. J. Clin. Endocrinol. Metab..

[18] Meadows A.T., Friedman D.L., Neglia J.P., Mertens A.C., Donaldson S.S., Stovall M., Hammond S., Yasui Y., Inskip P.D. (2009). Second neoplasms in survivors of childhoodcancer: Findings from the Childhood Cancer Survivor Study cohort. J. Clin. Oncol..

[19] Veiga L.H., Holmberg E., Anderson H., Pottern L., Sadetzki S., Adams M.J., Sakata R., Schneider A.B., Inskip P., Bhatti P. (2016). Thyroid Cancer after Childhood Exposure to External Radiation: An Updated Pooled Analysis of 12 Studies. Radiat. Res..

[20] Weiss W. (2018). Chernobyl thyroid cancer: 30 years of follow-up overview. Radiat. Prot. Dosim..

[21] Zimmermann M.B., Galetti V. (2015). Iodine intake as a risk factor for thyroid cancer: A comprehensive review of animal and human studies. Thyroid Res..

[22] Gardner D.G., Shoback D. (2012). Hormones and hormone action. Green Spans Basic and Clinical Endocrinology.

[23] Boelaert K., Horacek J., Holder R.L., Watkinson J.C., Sheppard M.C., Franklyn J.A. (2006). Serum thyrotropin concentration as a novel predictor of malignancy in thyroid nodules investigated by fine-needle aspiration. J. Clin. Endocrinol. Metab..

[24] Haugen B.R., Alexander E.K., Bible K.C., Doherty G.M., Mandel S.J., Nikiforov Y.E., Pacini F., Randolph G.W., Sawka A.M., Schlumberger M. (2016). 2015 American Thyroid Association Management Guidelines for Adult Patients with Thyroid Nodules and Differentiated Thyroid Cancer: The American Thyroid Association Guidelines Task Force on Thyroid Nodules and Differentiated Thyroid Cancer. Thyroid.

[25] Lyshchik A., Drozd V., Demidchik Y., Reiners C. (2005). Diagnosis of thyroid cancer in children: Value of grayscale and power doppler US. Radiology.

[26] Essenmacher A.C., Joyce P.H., Kao S.C., Epelman M., Pesce L.M., D’Alessandro M.P., Sato Y., Johnson C.M., Podberesky D.J. (2017). Sonographic Evaluation of Pediatric Thyroid Nodules. Radiographics.

[27] Gupta A., Ly S., Castroneves L.A., Frates M.C., Benson C.B., Feldman H.A., Wassner A.J., Smith J.R., Marqusee E., Alexander E.K. (2013). A standardized assessmentof thyroid nodules in children confirms highercancer prevalence than in adults. J. Clin. Endocrinol. Metab..

[28] Al Nofal A., Gionfriddo M.R., Javed A., Haydour Q., Brito J.P., Prokop L.J., Pittock S.T., Murad M.H. (2016). Accuracy ofthyroid nodule sonography for the detection of thyroid cancerin children: Systematic review and meta-analysis. Clin. Endocrinol..

[29] Koltin D., O’Gorman C.S., Murphy A., Ngan B., Daneman A., Navarro O.M., García C., Atenafu E.G., Wasserman J.D., Hamilton J. (2016). Pediatric thyroidnodules: Ultrasonographic characteristics and inter-observervariability in prediction of malignancy. J. Pediatr. Endocrinol. Metab..

[30] Richman D.M., Benson C.B., Doubilet P.M., Peters H.E., Huang S.A., Asch E., Wassner A.J., Smith J.R., Cherella C.E., Frates M.C. (2018). Thyroid Nodules in Pediatric Patients: Sonographic Characteristics and Likelihood of Cancer. Radiology.

[31] Cantisani V., Lodise P., Grazhdani H., Mancuso E., Maggini E., Di Rocco G., D’Ambrosio F., Calliada F., Redler A., Ricci P. (2014). Ultrasound elastography in the evaluation of thyroid pathology. Current status. Eur. J. Radiol..

[32] Zhao C.K., Xu H.X. (2019). Ultrasound elastography of the thyroid: Principles and current status. Ultrasonography.

[33] Cunha G.B., Marino L.C.I., Yamaya A., Kochi C., Monte O., Longui C.A., Cury A.N., Fleury E.D.F.C. (2019). Elastography for the evaluation of thyroid nodules in pediatric patients. Radiol. Bras..

[34] Pawluś A., Sokołowska-Dąbek D., Szymańska K., Inglot M.S., Zaleska-Dorobisz U. (2015). Ultrasound elastography: Review of techniques and its clinical applications in pediatrics—Part 1. Adv. Clin. Exp. Med..

[35] Rago T., Vitti P. (2008). Role of thyroid ultrasound in the diagnostic evaluation of thyroid nodules. Best Pract. Res. Clin. Endocrinol. Metab..

[36] Yurttutan N., Gungor G., Bilal N., Kizildag B., Baykara M., Sarica M.A. (2016). Interpretation of thyroid glands in a group of healthy children: Real-time ultrasonography elastography study. J. Pediatr. Endocrinol. Metab..

[37] Palabıyık F.B., İnci E., PapatyaÇakır E.D., Hocaoğlu E. (2019). Evaluation of Normal Thyroid Tissue and Autoimmune Thyroiditis in Children Using Shear Wave Elastography. J. Clin. Res. Pediatr. Endocrinol..

[38] Borysewicz-Sanczyk H., Dzieciol J., Sawicka B., Bossowski A. (2016). Practical application ofelastography in the diagnosis of thyroid nodules in children and adolescents. Horm. Res. Paediatr..

[39] Partyka K.L., Huang E.C., Cramer H.M., Chen S., Wu H.H. (2016). Histologic and clinical follow-up of thyroidfine-needle aspirates in pediatric patients. Cancer Cytopathol..

[40] Cibas E.S., Ali S.Z. (2009). NCI Thyroid FNA State of the Science Conference. The Bethesda System for Reporting Thyroid Cytopathology. Am. J. Clin. Pathol..

[41] Cherella C.E., Angell T.E., Richman D.M., Frates M.C., Benson C.B., Moore F.D., Barletta J.A., Hollowell M., Smith J.R., Alexander E.K. (2019). Differences in Thyroid Nodule Cytology and Malignancy Risk between Children and Adults. Thyroid.

[42] Giovanella L., Avram A.M., Iakovou I., Kwak J., Lawson S.A., Lulaj E., Luster M., Piccardo A., Schmidt M., Tulchinsky M. (2019). EANM practice guideline/SNMMI procedure standard for RAIU and thyroid scintigraphy. Eur. J. Nucl. Med. Mol. Imaging.

[43] Kresnik E., Gallowitsch H.J., Mikosch P., Stettner H., Igerc I., Gomez I., Kumnig G., Lind P. (2003). Fluorine-18-fluorodeoxyglucose positron emission tomography in the preoperative assessment of thyroid nodules in an endemic goiter area. Surgery.

[44] Kim J.M., Ryu J.S., Kim T.Y., Kim W.B., Kwon G.Y., Gong G., Moon D.H., Kim S.C., Hong S.J., Shong Y.K. (2007). 18F-fluorodeoxyglucose positron emission tomography does not predict malignancy in thyroid nodules cytologically diagnosed as follicular neoplasm. J. Clin. Endocrinol. Metab..

[45] Sebastianes F.M., Cerci J.J., Zanoni P.H., Soares J., Chibana L.K., Tomimori E.K., de Camargo R.Y., Izaki M., Giorgi M.C., Eluf-Neto J. (2007). Role of 18F-fluorodeoxyglucose positron emission tomography in preoperative assessment of cytologically indeterminate thyroid nodules. J. Clin. Endocrinol. Metab..

[46] Hales N.W., Krempl G.A., Medina J.E. (2008). Is there a role for fluorodeoxyglucose positron emission tomography/computed tomography in cytologically indeterminate thyroid nodules?. Am. J. Otolaryngol..

[47] Wang N., Zhai H., Lu Y. (2013). Is fluorine-18 fluorodeoxyglucose positron emission tomography useful for the thyroid nodules with indeterminate fine needle aspiration biopsy? A meta-analysis of the literature. J. Otolaryngol. Head Neck Surg..

[48] Vriens D., Adang E.M., Netea-Maier R.T., Smit J.W., de Wilt J.H., Oyen W.J., de Geus-Oei L.F. (2014). Cost-effectiveness of FDG-PET/CT for cytologically indeterminate thyroid nodules: A decision analytic approach. J. Clin. Endocrinol. Metab..

[49] Piccardo A., Puntoni M., Treglia G., Foppiani L., Bertagna F., Paparo F., Massollo M., Dib B., Paone G., Arlandini A. (2016). Thyroid nodules with indeterminate cytology: Prospective comparison between 18F-FDG-PET/CT, multiparametric neck ultrasonography, 99mTc-MIBI scintigraphy and histology. Eur. J. Endocrinol..

[50] Giovanella L., Suriano S., Maffioli M., Ceriani L., Spriano G. (2010). (99m)Tc-sestamibi scanning in thyroid nodules with nondiagnostic cytology. Head Neck.

[51] Leidig-Bruckner G., Cichorowski G., Sattler P., Bruckner T., Sattler B. (2012). Evaluation of thyroid nodules—Combined use of 99mTc-methylisobutylnitrile scintigraphy and aspiration cytology to assess risk of malignancy and stratify patients for surgical or nonsurgical therapy—A retrospective cohort study. Clin. Endocrinol..

[52] Saggiorato E., Angusti T., Rosas R., Martinese M., Finessi M., Arecco F., Trevisiol E., Bergero N., Puligheddu B., Volante M. (2009). 99mTc-MIBI Imaging in the Presurgical Characterization of Thyroid Follicular Neoplasms: Relationship to Multidrug Resistance Protein Expression. J. Nucl. Med..

[53] Campennì A., Siracusa M., Ruggeri R.M., Laudicella R., Pignata S.A., Baldari S., Giovanella L. (2017). Differentiating malignant from benign thyroid nodules with indeterminate cytology by 99mTc-MIBI scan: A new quantitative method for improving diagnostic accuracy. Sci. Rep..

[54] Hoang J.K., Branstetter B.F., Gafton A.R., Lee W.K., Glastonbury C.M. (2013). Imaging of thyroid carcinoma with CT and MRI: Approaches to common scenarios. Cancer Imaging.

[55] Lubin J.H., Adams M.J., Shore R., Holmberg E., Schneider A.B., Hawkins M.M., Robison L.L., Inskip P.D., Lundell M., Johansson R. (2017). Thyroid Cancer Following Childhood Low-Dose Radiation Exposure: A Pooled Analysis of Nine Cohorts. J. Clin. Endocrinol. Metab..

[56] Hempel J.M., Kloeckner R., Krick S., Pinto dos Santos D., Schadmand-Fischer S., Boeßert P., Bisdas S., Weber M.M., Fottner C., Musholt T.J. (2016). Impact of combined FDG-PET/CT and MRI on the detection of local recurrence and nodal metastases in thyroid cancer. Cancer Imaging.

